# Minimizing atelectasis formation during general anaesthesia—oxygen washout is a non-essential supplement to PEEP

**DOI:** 10.1080/03009734.2017.1294635

**Published:** 2017-04-24

**Authors:** Erland Östberg, Udo Auner, Mats Enlund, Henrik Zetterström, Lennart Edmark

**Affiliations:** a Department of Anaesthesia and Intensive Care, Västerås and Köping Hospital, Västerås, Sweden;; b Department of Radiology, Västerås Hospital, Västerås, Sweden;; c Centre for Clinical Research, Västerås, Sweden;; d Department of Surgical Sciences, Anaesthesiology and Intensive Care, Uppsala University, Uppsala, Sweden

**Keywords:** Atelectasis, computed tomography, general anaesthesia, oxygenation, PEEP, protective ventilation, ventilator settings

## Abstract

**Background:**

Following preoxygenation and induction of anaesthesia, most patients develop atelectasis. We hypothesized that an immediate restoration to a low oxygen level in the alveoli would prevent atelectasis formation and improve oxygenation during the ensuing anaesthesia.

**Methods:**

We randomly assigned 24 patients to either a control group (*n* = 12) or an intervention group (*n* = 12) receiving an oxygen washout procedure directly after intubation. Both groups were, depending on body mass index, ventilated with a positive end-expiratory pressure (PEEP) of 6–8 cmH_2_O during surgery. The atelectasis area was studied by computed tomography before emergence. Oxygenation levels were evaluated by measuring blood gases and calculating estimated venous admixture (EVA).

**Results:**

The atelectasis areas expressed as percentages of the total lung area were 2.0 (1.5–2.7) (median [interquartile range]) and 1.8 (1.4–3.3) in the intervention and control groups, respectively. The difference was non-significant, and also oxygenation was similar between the two groups. Compared to oxygenation before the start of anaesthesia, oxygenation at the end of surgery was improved in the intervention group, mean (SD) EVA from 7.6% (6.6%) to 3.9% (2.9%) (*P* = .019) and preserved in the control group, mean (SD) EVA from 5.0% (5.3%) to 5.6% (7.1%) (*P* = .59).

**Conclusion:**

Although the oxygen washout restored a low pulmonary oxygen level within minutes, it did not further reduce atelectasis size. Both study groups had small atelectasis and good oxygenation. These results suggest that a moderate PEEP alone is sufficient to minimize atelectasis and maintain oxygenation in healthy patients.

## Introduction

Atelectasis formation during anaesthesia is the result of airway closure and is dependent on both the oxygen concentration in the lungs and time (1).

Preoxygenation before anaesthesia induction is a safety measure that is applied to increase the apnoea tolerance time in cases of expected or unexpected difficult airway. However, a high oxygen content in the lung units may lead to gas absorption and subsequent alveolar collapse (2). As soon as the trachea is successfully intubated, the initial desirable effect of preoxygenation changes to become both unnecessary and potentially harmful (3). Previous investigations have demonstrated that atelectasis formation occurs early after induction and intubation (4), and contributes to impaired oxygenation and, possibly, postoperative complications (5). For safety reasons, the avoidance of preoxygenation cannot be recommended, but rapidly eliminating its effect once the airway is secured might minimize the time-frame during which the alveoli are exposed to the ‘oxygen threat’ (6).

We hypothesized that an immediate restoration to a low oxygen content would protect the lungs from alveolar collapse and thereby decrease atelectasis formation and improve oxygenation during anaesthesia.

## Material and methods

This prospective, evaluator-blinded, randomized controlled trial was approved by the Regional Ethics Committee in Uppsala, Sweden on 28 April 2014 (ref: Dnr 2012/335) and registered with the ClinicalTrials.gov (NCT02216006). Written informed consent was obtained from all participating patients.

### Study population

We included 24 patients with American Society of Anesthesiologists (ASA) physical status 1–2, aged 40–75 years and with a body mass index (BMI) less than 30 kg/m^2^, who were scheduled for elective orthopaedic day surgery at Köping Hospital, Sweden. Only patients subjected to extremity surgery in a supine position were included. Patients were excluded if they had any significant obstructive pulmonary disease, heart failure, ischaemic heart disease, anaemia (haemoglobin <100 g/L), or peripheral oxygen saturation (SpO_2_) less than 94% when breathing room air. Patients with a known or expected difficult airway, as well as smokers and ex-smokers with a history of more than six pack years, were excluded.

### Randomization

All patients were assessed for eligibility by the corresponding author. Using a sealed-envelope technique, they were randomly assigned to either an intervention group or a control group.

### Anaesthesia and monitoring

No patient received any premedication. Prior to the start of anaesthesia, an arterial catheter was placed in the radial artery under local anaesthesia. Monitoring was performed using the Philips IntelliVue MP anaesthesia monitor (Philips Medizin Systeme, Boeblingen, Germany) and included invasive arterial blood pressure and continuous three-lead electrocardiogram. Peripheral oxygen saturation was measured with the Masimo Rad-5 (Masimo Corporation, Irvine, CA, USA). The Datex-Ohmeda S/5 Avance (GE Healthcare, Datex-Ohmeda, Madison, WI, USA) was used for ventilation along with a D-lite sidestream adapter (GE Healthcare Finland Oy, Helsinki, Finland), with ports for spirometry and gas sampling at the Y-piece.

The patients were placed in a supine position with a 15–20-degree head-up tilt and were preoxygenated with an inspired oxygen fraction (F_I_O_2_) of 1.0 for 3 min. Target-controlled infusions (TCI) of propofol and remifentanil (Injectomat TIVA Agilia, Fresenius Kabi AB, Uppsala, Sweden) were used to induce anaesthesia. To facilitate tracheal intubation, 0.5 mg/kg rocuronium was administered 1 min after induction followed by bag mask ventilation with an F_I_O_2_ of 1.0 for 2 min. Correct tracheal intubation was confirmed through auscultation (during manual ventilation with an F_I_O_2_ of 1.0) and visualization of the return of carbon dioxide (CO_2_) on the anaesthesia monitor. To ensure uniformity, all 24 patients were anaesthetized by the same two anaesthetists (authors E.Ö. and L.E.).

Anaesthesia was maintained by TCI infusions of propofol and remifentanil. The patients’ mean arterial blood pressure was maintained above 60 mmHg, with small doses of intravenous phenylephrine or ephedrine administered as needed. The patients were ventilated using a volume-controlled mode and a circle system. The tidal volumes were set to 7 mL/kg ideal body weight (IBW) (7) and positive end-expiratory pressure (PEEP) levels of 6 or 8 cmH_2_O, depending on the BMI (the higher level if the BMI was equal to 25 kg/m^2^ or above). Fresh gas flow, with a fractional oxygen content of 0.40–0.45, was set to 1 L/min, aiming for an F_I_O_2_ between 0.30 and 0.35. The respiratory rate was set to 9–11 per minute, aiming for an end-tidal carbon dioxide partial pressure of 5 kPa.

### Intervention

As soon as correct tracheal tube placement was confirmed, the control group was ventilated with the settings described above. The intervention group was first subjected to an oxygen washout manoeuvre consisting of a pre-set volume-controlled ventilation with a respiratory rate of 10 per minute and using: (1) a high fresh gas flow (10 L/min); (2) a low oxygen content, i.e. air; (3) large tidal volumes (15 mL/kg IBW); and (4) a PEEP of 10 cmH_2_O. Using the expired oxygen content as a measurement for low lung oxygen content, the procedure was considered complete when the end-tidal oxygen concentration (EtO_2_) had decreased to 25%. As soon as this goal was achieved, the ventilator settings were changed to the settings described for the control group, with the same F_I_O_2_ of 0.30–0.35.

### Computed tomography scan

After the completion of surgery, the tracheal tube was clamped at end-expiration. The anaesthesia machine was disconnected, and the patients were switched to a portable ventilator, the Vivo 50 (Breas Medical AB, Mölnlycke, Sweden), which maintained the same PEEP and ventilation settings. A computed tomography (CT) scan was performed in the Radiology Department, located directly below the operating room. We used a GE LightSpeed VCT XTe (GE Healthcare, Waukesha, WI, USA) to study the atelectasis area. Patients were placed in a supine position, and a frontal scout view was obtained at end-expiration to determine the location of the diaphragm. A basal single-sliced transverse scan with a thickness of 5 mm was then performed 5–10 mm above the right diaphragm dome.

To calculate the atelectasis area, we first used the workstation software (AW Server 2.0 GE Healthcare, Chicago, IL, USA) to accurately delineate the contours of the lungs. Second, using a separate region of interest (ROI) technique, the atelectasis region was outlined ventrally with some margin beyond the radiological appearance of haziness in the lung parenchyma. Vascular structures larger than 3 mm in diameter were manually excluded when drawing the atelectasis ROI, and the pulmonary hilus vessels were excluded from the lung ROI. Finally, we used the histogram functional view to identify the actual atelectasis area, which was defined as –100 to +100 Hounsfield units (HU) (8). Atelectasis was expressed in cm^2^ and as the percentage of the whole lung area in the basal slice. All CT scans were assessed by the same radiologist, who was blinded to the group assignment.

### Arterial blood gases

Arterial blood gas samples were drawn from the arterial catheter while each patient remained in a head-up tilt position of 15–20 degrees. The first sample was drawn before anaesthesia, when the patient was breathing room air, and the second blood sample was drawn at the end of surgery with an F_I_O_2_ of 0.30–0.35, just prior to ventilator exchange. The blood gases were analysed immediately after sampling using the Radiometer ABL800 Flex (Radiometer Medical, Brønshøj, Denmark).

### Oxygenation

Because mixed venous blood was not collected, we used the estimated venous admixture (EVA) as the primary measure of oxygenation. This value was calculated from the arterial blood gases, as described by Zetterström (9), and with the assumption that the arteriovenous oxygen content difference was 40 mL/L. Because of its widespread use, we also calculated the arterial oxygen partial pressure (PaO_2_)/F_I_O_2_ (P/F) ratio to further illustrate any changes in oxygenation during anaesthesia.

### Statistical analysis

In accordance with previous studies, we expected the study subjects in the control group to exhibit atelectasis areas of at least 4% of the lung area (8). With a standard deviation of approximately 1.7 percentage points, we calculated that a sample size of 24 patients would achieve 80% power in detecting a difference of 50% in atelectasis with *P* = .05 between the groups. Because the outcome data were not expected to be normally distributed, comparisons between the groups were performed using the Mann–Whitney *U* test or Fischer’s exact test in the case of binary outcomes. Related samples within each group were analysed using the Wilcoxon signed-rank test. For all tests, a two-sided *P* value of <.05 was considered significant. Statistical analyses were performed using IBM SPSS Statistics version 22 (IBM Corporation, New York, NY, USA).

## Results

**Figure 1.**
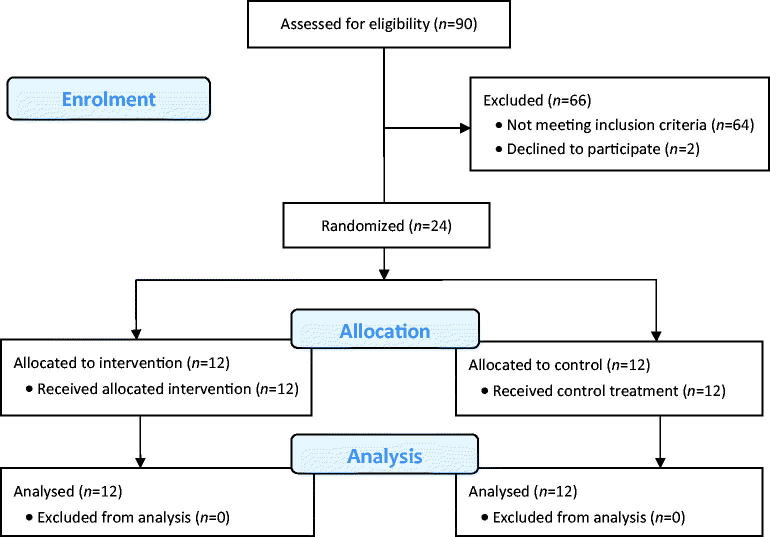
Consort diagram of study.

To achieve our sample size, in total 90 patients scheduled for day surgery were assessed for eligibility between September 2014 and May 2015. Of them, 64 did not meet the inclusion criteria, and two patients declined to participate. The remaining 24 patients were randomized for treatment. All received the allocated treatment, and all patients were analysed (Figure 1). Data described below are presented as mean (SD), unless stated otherwise.

Patient characteristics and baseline physiological data did not differ between the groups (Table 1). For all patients, mask ventilation ended at 6.5 min (0.6 min) after the start of preoxygenation. All patients were successfully intubated within 65 s thereafter.

**Table 1.** Patient characteristics and baseline physiological data in the two study groups.

	Oxygen washout group (*n* = 12)	Control group (*n* = 12)
Male/female	7/5	2/10
Age (years)	53 (9)	57 (9)
BMI (kg/m^2^)	25.2 (2.2)	23.8 (1.6)
ASA grade; 1/2	7/5	10/2
Haemoglobin (g/L)	140 (12)	135 (8)
SpO_2_ (%)	98 (1)	98 (1)
PaCO_2_ (kPa)	5.1 (0.5)	5.2 (0.3)
PaO_2_ (kPa)	11.9 (1.9)	12.4 (1.6)
SaO_2_ (%)	97.3 (1.4)	97.6 (1.4)
EVA (%)	7.6 (6.6)	5.0 (5.3)
P/F ratio (kPa)	56 (9)	59 (7)

Values are numbers or means (SD). PaCO_2_, PaO_2_, SaO_2_, and EVA were analysed and calculated from the first blood gas sample obtained before induction and with all patients breathing room air.

ASA: physical status according to American Society of Anesthesiologists; BMI: body mass index; EVA: estimated venous admixture; P/F ratio: PaO_2_ divided by inspired oxygen fraction (F_I_O_2_); PaCO_2_: arterial carbon dioxide partial pressure; PaO_2_: arterial oxygen partial pressure; SaO_2_: arterial oxygen saturation; SpO_2_: peripheral oxygen saturation.

**Figure 2.**
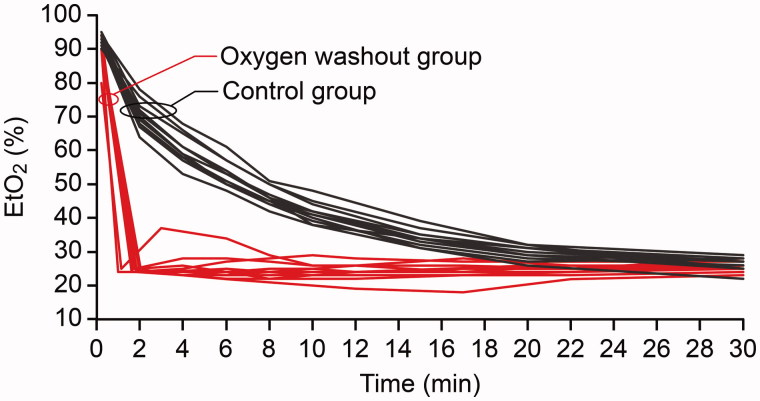
End-tidal oxygen concentration (EtO_2_) and time during anaesthesia after completed preoxygenation, induction, and intubation. Oxygen washout group, *t*_0_ = start of oxygen washout manoeuvre. Control group, *t*_0_ = start of maintenance ventilation using a fresh gas flow of 1 L/min with a fractional oxygen content of 0.40.

In the intervention group, the time from start to completion of the oxygen washout manoeuvre, i.e. the time required for the EtO_2_ to reach 25% or below, was 1.6 min (0.4 min) (Figure 2). No desaturation (SpO_2_ <94%) was recorded in any subject during the procedure, and no patient exhibited peak inspiratory pressures above 30 cmH_2_O. The control group receiving standard treatment required 20 min (3.3 min) to reach the targeted F_I_O_2_ of 0.30–0.35. The corresponding EtO_2_ levels have been depicted in Figure 2.

**Figure 3.**
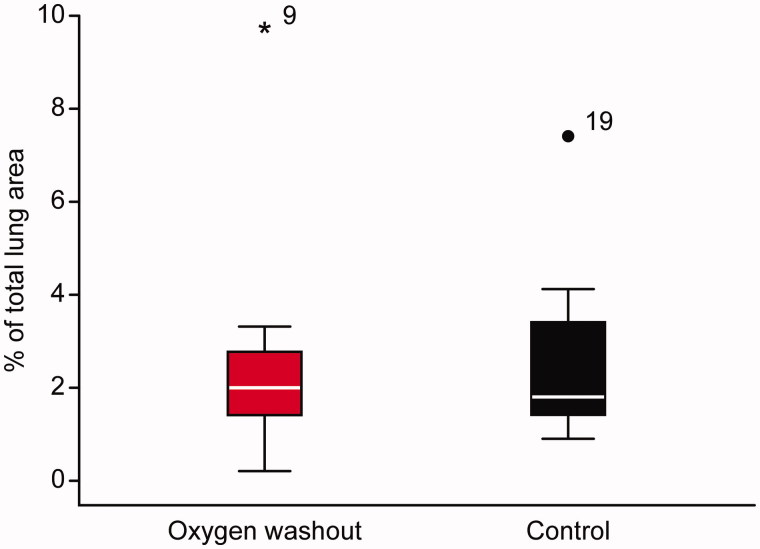
Atelectasis area sizes, as expressed by percentages of total lung area in the two groups. Data presented as median, interquartile range (box) and range (whiskers), *P* = .98. Dots represent outliers, one in each group (subjects no. 9 and no. 19). CT scans to study atelectasis area sizes were performed 5–10 mm above the right diaphragm dome at the end of surgery.

No difference in the atelectasis area size was found between the groups. The calculated areas expressed as percentages of the total lung area were median (interquartile range) 2.0 (1.3–2.8) and 1.8 (1.3–3.6) in the intervention and control groups, respectively, *P* = .98 (Figure 3). Additionally, there was no difference between the groups with regard to oxygenation or physiological data at the time of the second blood gas sampling (Table 2). The duration of surgery in the intervention group was longer than in the control group. Consequently, the duration of anaesthesia was longer (Table 2), and the time for CT scan (min after start of preoxygenation) also differed between the groups: 110 min (28 min) and 88 min (23 min) for the intervention and control groups, respectively (*P* = .039).

**Table 2.** Physiological data and duration of anaesthesia in the two study groups, at the time of the second blood gas sample. Both groups were ventilated with an F_I_O_2_ of 0.30–0.35.

	Oxygen washout group (*n* = 12)	Control group (*n* = 12)
SpO_2_ (%)	98 (1)	98 (1)
PaCO_2_ (kPa)	5.4 (0.3)	5.5 (0.3)
PaO_2_ (kPa)	19.9 (3.3)	20.1 (4.2)
SaO_2_ (%)	99.3 (0.5)	99 .0 (1.6)
EVA (%)	3.9 (2.9)	5.6 (7.1)
P/F ratio (kPa)	64 (10)	61 (13)
Duration of anaesthesia (min)	90 (26)	68 (23)^a^

Values are means (SD). PaCO_2_, PaO_2_, SaO_2_, and EVA were analysed and calculated from the second blood gas sample.

a Duration of anaesthesia, *P* = .039, Mann–Whitney *U* test.

EVA: estimated venous admixture; P/F ratio: PaO_2_ divided by inspired oxygen fraction (F_I_O_2_); PaCO_2_: arterial carbon dioxide partial pressure; PaO_2_: arterial oxygen partial pressure; SaO_2_: arterial oxygen saturation; SpO_2_: peripheral oxygen saturation.

In the intervention group, oxygenation improved from the first (before induction) to the second blood gas sample (end of surgery). This was illustrated by a decline in EVA from 7.6% (6.6%) to 3.9% (2.9%) (*P* = .019) accompanied by an increase in P/F ratio from 56 kPa (9 kPa) to 64 kPa (10 kPa) (*P* = .005). The control group exhibited preserved oxygenation at the end of the surgery (Table 2) compared with oxygenation before anaesthesia (Table 1).

Apart from two outliers, further described in the Discussion, we observed no patient complications during the study.

## Discussion

### Main findings

In this randomized controlled trial that examined patients ventilated with a moderate PEEP, early restoration to low alveolar oxygen levels after preoxygenation and intubation did not affect atelectasis size as studied at the end of surgery. The atelectasis area was small in both groups, which likely contributed to the good oxygenation indices observed.

**Figure 4.**
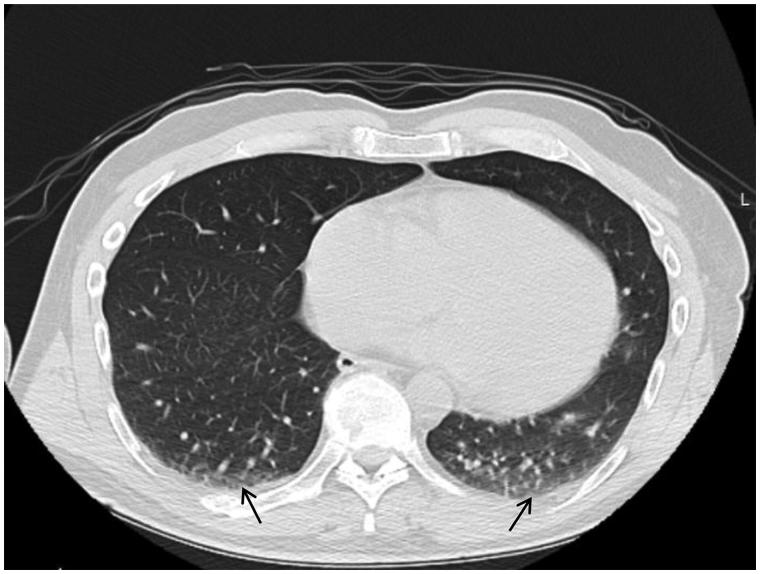
CT scan of one of the study subjects at the end of surgery. The arrows indicate small areas of atelectasis formation. In this case, 1.9% of the total lung area was affected, and this value corresponded to the median of all subjects in the study.

To our knowledge, no other research group has studied the atelectasis area at the end of surgery in anaesthetized subjects ventilated with PEEP. In the current trial, the median atelectasis area as a percentage of the total lung area was 1.9 for all patients (Figure 4). This value is approximately half that of areas described in previous studies shortly after intubation (1,8).

As explained by Dantzker et al. (10) and later Joyce et al. (11), the mechanisms by which atelectasis forms during general anaesthesia primarily involve airway closure due to decreased end-expiratory lung volume and subsequent gas absorption because of high alveolar oxygen content after preoxygenation. The process is dependent on both time and oxygen concentration in the alveoli (12). The aim of the current study was to investigate the effects of partially eliminating one of the factors contributing to atelectasis formation, i.e. high initial alveolar oxygen content, by using a simple and low-risk intervention that could be used for all patients. Thus, the aim was atelectasis *prevention* by rapidly restoring a low oxygen content in the lung, instead of treating *already existing* atelectasis using a more complex recruitment manoeuvre with higher peak pressures. Furthermore, we aimed to investigate only the effect of oxygen washout and otherwise adhere to ‘best practice’ recommendations, i.e. tidal volumes of 7 mL/kg and a PEEP level of 6–8 cmH_2_O (1). In order to minimize confounding effects from surgery per se, we chose a study group undergoing low-risk orthopaedic surgery.

Apart from the initial oxygen washout manoeuvre and the use of PEEP, we did not take any measures to prevent or treat atelectasis. One possible explanation for the small atelectasis area observed is that the PEEP level maintained during anaesthesia actually recruited collapsed lung units that formed during and after preoxygenation and intubation. A so-called ‘PEEP recruitment’ has been described in previous studies, although with a higher PEEP level of 10 cmH_2_O (4,13,14). In the study by Brismar et al. (4), the effect of PEEP was observed within 5 min of its application, indicating that the main PEEP effect occurs shortly after its application in healthy lungs. Whether lower PEEP levels of 6–8 cmH_2_O are sufficient to open up already formed atelectasis is unknown. However, in the current study, we assume that such a recruitment effect was operative in equal amounts in both groups, although likely to a lesser degree because of lower peak airway pressures.

Regarding oxygenation, no statistically significant difference was found between the groups. Contrary to the observation that impaired oxygenation usually accompanies general anaesthesia, no such deterioration was observed in any of the study groups, as clearly demonstrated by the values of both the EVA and P/F ratio at the end of surgery (Table 2). In fact, the intervention group even demonstrated an improved oxygenation compared with oxygenation before anaesthesia induction. Although not statistically significant, the intervention group had a higher EVA than did the control group before anaesthesia induction, and also the P/F ratio was lower in the intervention group (Table 1). An increased venous admixture prior to induction is likely explained by intermittent airway closure in the awake state. Assuming proper preoxygenation, i.e. F_I_O_2_ of 1.0 for at least 3 min before the start of anaesthesia, most lung units would be filled with pure oxygen. Following anaesthesia induction, the end-expiratory lung volume will fall (15,16) and further increase airway closure (17,18). Some preoxygenated alveoli will then be ‘trapped’ distal to an entirely closed or semi-closed airway and will thus be particularly prone to atelectasis formation due to gas absorption (10,12). It is thus noticeable that the intervention group had less venous admixture (less shunt and/or less impact from areas in the lungs with a low alveolar ventilation [V_A_] to lung perfusion [Q] ratio with shunt-like effect) at the end of surgery while still under general anaesthesia than while awake.

One could perhaps argue that the observed improvement in oxygenation was simply and entirely a consequence of the higher F_I_O_2_ used during anaesthesia. However, a more likely explanation is a combined effect of PEEP and increased F_I_O_2_, particularly in view of the longer duration of PEEP in this group. Furthermore, we cannot exclude the possibility that the oxygen washout performed in the intervention group really had the intended effect and thus shortened the exposure time of high oxygen concentration in susceptible lung units, thereby contributing to improved oxygenation and the minor atelectasis formation observed at the end of anaesthesia.

### Clinical implications

Despite large multicentre studies investigating the outcomes of intraoperative ventilation strategies, there is still conflicting evidence regarding the role of protective ventilation in general and PEEP in particular (19,20). More recent meta-analyses favour the addition of intermittent recruitment manoeuvres throughout anaesthesia to improve outcomes (21,22). However, whether this is necessary in every patient undergoing general anaesthesia is uncertain. The findings in the current study indicate that a moderate PEEP alone may be sufficient to maintain oxygenation and nearly prevent atelectasis in a vast majority of normal-weight, healthy patients undergoing low-risk surgery.

Regarding oxygen washout, this study shows that after preoxygenation and intubation, up to 20 minutes may be required to reach the intended oxygen level in a circle system (Figure 2), unless active measures are taken. An early oxygen washout will, within minutes, restore low pulmonary oxygen levels but seems to have no effect on atelectasis formation, provided an adequate PEEP level is used during subsequent anaesthesia.

### Study limitations

Open airways are a prerequisite for the oxygen washout to be effective. The manoeuvre was performed during (mean) 1.6 minutes immediately after intubation and with concomitant high PEEP and large tidal volumes. We believe that the vast majority of airways were open and accessible under these circumstances and time-frame. However, the exact speed by which atelectasis forms is unknown. Therefore, we cannot exclude the possibility that some alveoli had already collapsed, a fact that would influence the effectiveness of the manoeuvre and thereby the results of this study.

The other main limitation in this study was that we only performed one CT scan and that it was done at the end of surgery instead of directly after the intervention. The main reason for this was logistical considerations in our day surgery unit. Furthermore, a CT scan also before anaesthesia induction might have rendered more appropriate comparisons possible but would have increased the absorbed radiation dose and complicated the procedure. Because both study groups exhibited surprisingly small atelectasis, we seem to have underestimated the effect of using a moderate PEEP during surgery.

In addition, changing the ventilator and transporting the patients to the radiology unit have some risk of disturbing steady-state pulmonary conditions. Despite taking measures to avoid such disturbances, one patient in the control group experienced bronchospasm, which required the disconnection of the ventilator and suctioning of the airways. This event possibly explains the concomitant atelectasis area of 7.4%. Additionally, in the intervention group, one patient who was taking heavy antihypertensive medication experienced an episode of profound hypotension following induction, which may have contributed to the relatively large atelectasis area of 9.7% observed in this patient. Both outliers are depicted in Figure 3.

## Conclusion

In this randomized controlled trial, an early intervention after preoxygenation and intubation, to restore nitrogen levels and thus stabilizing the alveoli, had no effect on the amount of atelectasis at the end of surgery. However, the two groups were both ventilated with a moderate PEEP during anaesthesia and had good oxygenation as well as small atelectasis. It may be an intriguing possibility that even moderate PEEP levels have recruitment properties and may alone be sufficient to limit atelectasis formation in healthy lungs.
